# The cutaneous response to a mosquito bite is influenced by the diurnal rhythm

**DOI:** 10.1016/j.isci.2025.113666

**Published:** 2025-09-30

**Authors:** Hamidah Raduwan, Jinhee Park, Alejandro Marín-López, Mathias H. Skadow, Tse-Yu Chen, Richard A. Flavell, Albert C. Shaw, Ruth R. Montgomery, Erol Fikrig

**Affiliations:** 1 Section of Infectious Diseases, Department of Internal Medicine, School of Medicine, Yale University, New Haven, CT 06520, USA; 2 Department of Psychological Science and Neuroscience, Belmont University, Nashville, TN, USA; 3 Center of Research in Animal Health (CISA-INIA, CSIC), Algete-El Casar road, km. 8.1, Madrid, Spain; 4 Department of Immunobiology, School of Medicine, Yale University, New Haven, CT 06520, USA; 5 Department of Biomedical Sciences, School of Medicine, Mercer University, Savannah, GA 31404, USA; 6 Howard Hughes Medical Institute, Yale University, New Haven, CT, USA; 7 Section of Rheumatology, Department of Internal Medicine, School of Medicine, Yale University, New Haven, CT 06520, USA

**Keywords:** immunology, dermatology, biological sciences, genetics

## Abstract

The influence of the time of day on the cutaneous immune response to mosquito feeding is not well understood. *Aedes aegypti* mosquitoes feed on mice throughout the day, and a bloodmeal is most often obtained at times of day that are equivalent to dawn (ZT1) and dusk (ZT11). We observed that cells in the murine skin elicited more differentially expressed genes at ZT11 compared to ZT1. Additionally, we detected more immune cells in the skin at ZT11 in response to a mosquito bite. These results suggest that assessments of host responses to a mosquito bite and mosquito-borne infections may be influenced by the diurnal rhythm.

## Highlights

• The cutaneous immune response to mosquito bites varies depending on the time of day
• Mosquito bites at ZT11 induce greater transcriptomic changes compared to ZT1
• A higher infiltration of immune cells is observed at the bite site at ZT11 versus ZT1
• Distinct subsets of immune cells exhibit time-dependent sensitivity to mosquito bites

## Graphical abstract

**Figure:**
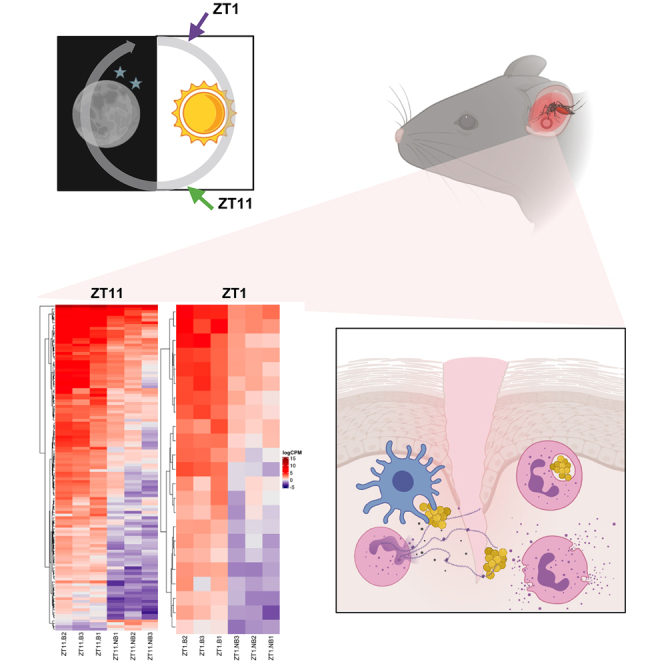

## Introduction

The skin is the largest organ and serves as a primary barrier against infection. Many biological properties of the skin display oscillation in activity and gene expression that are regulated by circadian and diurnal rhythm.^1^^,^^2^ During the period of activity, immune defenses on the skin are heightened, while during the period of rest, the skin undergoes repair and regeneration. The circadian clock controls the infiltration of dendritic cells,^3^ leukocytes,^4^^,^^5^ and neutrophil^6^ into lymphatic vessels, a process that is essential for many adaptive immune responses and relevant for vaccination and immunotherapies.^3^ Components of cell proliferation and wound-healing in the skin,^7^ as well as the oscillation of expression of antimicrobial peptides^8^ and interferon-stimulated genes,^9^ all of which are responsible for defense against pathogenic infection, are also subject to circadian regulation.

Regulation of circadian rhythm is controlled by a set of core-clock genes. *BMAL1* (*brain and muscle ARNT-like 1*) is a transcription factor that serves as a major regulator in the transcription/translation feedback loop of the biological clock. Rhythmic *BMAL1* expression is important for daily molecular oscillations of gene expression in many tissues such as the skin^10^ and liver.^11^ Disruption of the circadian rhythm has been linked to various infections and disease pathogenesis.^12^ Indeed, mice deficient in *BMAL1* exhibit greater viral replication and dissemination during infection,^13^^,^^14^ suggesting a role for circadian rhythms influencing antiviral functions.

Hematophagous mosquitoes are an important vector for many pathogens such as the arboviruses that transmit yellow fever, West Nile, Zika, dengue, and chikungunya viruses, and the *Plasmodium* parasites that cause malaria. Mosquitoes exhibited diurnal variation of locomotor/flight activity,^15^ host-seeking,^16^^,^^17^ biting/blood-feeding,^16^^,^^17^ mating,^16^^,^^17^^,^^18^ and oviposition.^19^ Defective circadian or diurnal cycling by means of either genetic or environmental disruption can result in reduced host-seeking behavior, reproductive rates, and adult survival and mating success.^16^^,^^17^ Other studies have shown a connection between the circadian rhythm and the propensity for *Anopheles gambiae* mosquitoes to be infected by *Plasmodium falciparum*.^20^

Mosquito saliva consists of a cocktail of pharmacologically active factors that have vasodilatory, anti-hemostatic, and immunomodulatory effects on the host which facilitate blood-feeding.^21^^,^^22^ These factors can also augment pathogen transmission during a bloodmeal. Numerous studies have shown that arboviruses or *Plasmodium*, introduced via a mosquito bite or via needle inoculation with the presence of mosquito saliva, lead to a higher pathogen load and more severe morbidity compared with needle inoculation of the pathogen without mosquito-derived factors.^23^^,^^24^ A recent study on humans suggests that mosquito bites trigger an innate immune response within hours, transitioning to an adaptive immune response two days post bite.^25^ Neutrophil influx and degranulation were observed as early as 4 h post-bite in humans^25^ and at 3 h in C57BL/6 immunocompetent mice.^26^ Strong T cell activation was noted later, at 2 days post-bite in humans, highlighting the shift to an adaptive immune response following a mosquito bite.^25^

The influence of diurnal rhythm on pathogen transmission facilitated by the mosquito bite has been largely understudied. A recent study showed an oscillatory pattern of gene expression in the salivary glands of *Anopheles* mosquitoes, and transmission of *Plasmodium berghei* to mice was influenced by the coordination of the circadian rhythm between the parasite, vector, and the host.^27^ In turn, host circadian rhythms can also be disrupted upon infection.^28^ Immune functions of the host skin, such as the expression of some immune genes, and migration of immune cells to and from the skin, exhibited a daily rhythmicity that is dependent on diel. In turn, the immune functions of the host skin can also be influenced by a mosquito bite. In this study, we found that diurnal rhythm influences the host immune response to a mosquito bite.

## Results

**Figure 1:**
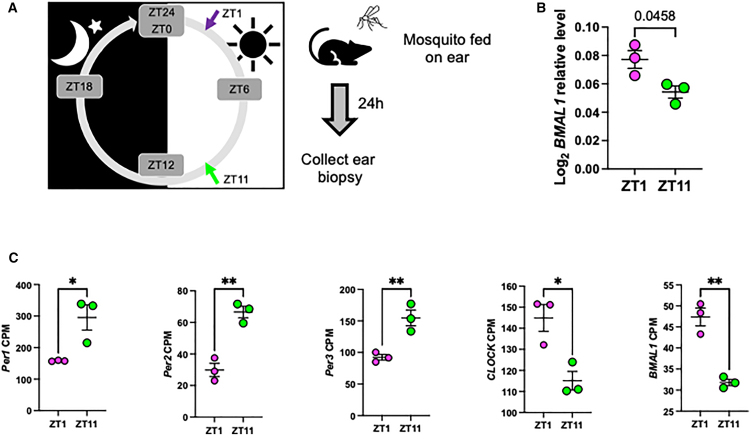
Murine ear biopsies were collected from mice after mosquito bite and assessed by RNA-sequencing (A) Ear biopsies were collected from mice 24 h after being bitten by *Aedes aegypti* mosquitos at the indicated time points (*n* = 3/group; magenta for ZT1 and green for ZT11). (B) *BMAL1*-relative expression of mice ear was verified to have high expression at ZT1 and low expression at ZT11 before samples are sent for RNA-sequencing. Statistics was performed using unpaired Welch’s *t* test; error bars represent standard error of the mean (sem). (C) Counts per million (CPM) value of *Per1*, *Per2*, *Per3*, *CLOCK*, and *BMAL1* after normalization of RNA-sequencing samples verified that the samples have good rhythmicity. Statistics was performed using unpaired Welch’s *t* test; ∗*p* < 0.05 and ∗∗*p* < 0.01; error bars represent standard error of the mean (SEM).

To study the effect of diurnal rhythm on eliciting differential cutaneous gene expression, we examined the transcriptional changes occurring in the skin upon mosquito bite at different times of day. Approximately 20 *Aedes aegypti* mosquitoes were allowed to bite mice on the ear at two timepoints, zeitgeber time (ZT)1 and ZT11, corresponding to dawn and dusk, respectively. Twenty-four hours later, biopsies from the mosquito bite sites were collected, and transcriptomic analyses were performed. Biopsies from mice that did not receive a mosquito bite served as controls (Figure 1A). To confirm that the mice had a robust diurnal rhythm in the skin, we quantified the expression of one of the core-clock genes; *BMAL1*. *BMAL1* expression was shown to peak at ZT1 and reached basal level at ZT11.^29^^,^^30^ Consistently, *BMAL1* RNA expression of the non-bitten tissue biopsies were confirmed to have high expression of *BMAL1* at ZT1 and low expression at ZT11^29^^,^^30^ (Figure 1B). Additionally, we examined the counts-per-million reads of some core-clock genes, *Per1*, *Per2*, *Per3*, *CLOCK*, and *BMAL1*, after normalization of the RNA-sequencing reads and showed that their expression followed the observed expression level as published elsewhere^29^^,^^30^ (Figure 1C). This demonstrated that the mice had a diurnal rhythm of gene expression in the current laboratory setting.

### Mosquito bite elicited a higher number of differentially expressed genes at ZT11

**Figure 2:**
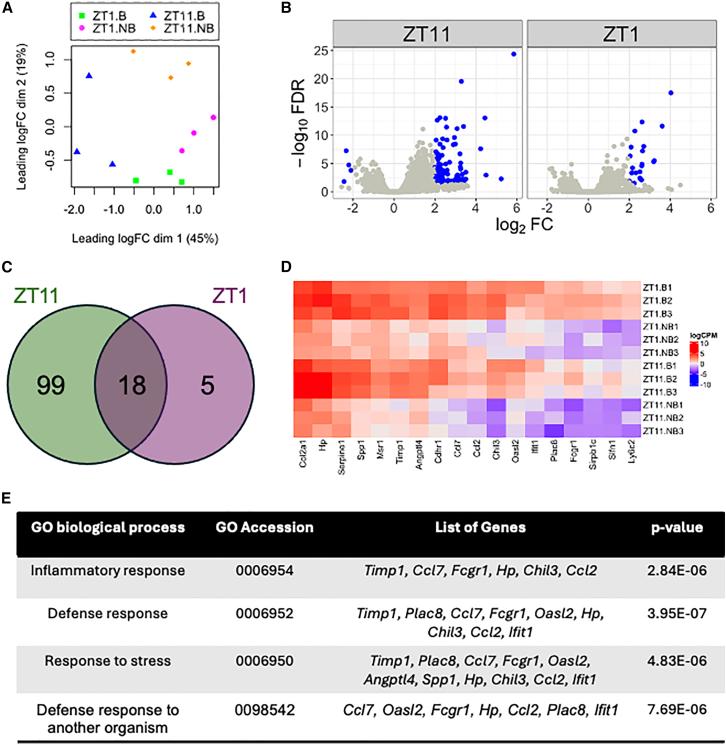
Murine skin showed differential gene expression upon mosquito bite (A) Multidimensional scaling plot showed clustering of samples when comparing no mosquito bite (NB) and mosquito bite (B) group for both zeitgeber time 11 (ZT11) and 1 (ZT1). (B) Volcano plot depicts expression of all genes (gray). Differentially expressed genes that meet the cutoff of ≤0.05 false discovery rate (FDR) and ≥ 2-fold log_2_FC are plotted in blue. (C) Venn diagram comparing the list of genes from ZT11 (green) and ZT1 (pink) showed that there are 18 genes that overlap between these two timepoints. (D) Heatmap depicting log_2_CPM for genes that are overlapping between ZT11 and ZT1. (E) GO-enrichment analysis for biological processes highlighted that the common genes are involved in inflammatory response, defense response, response to stress, and defense response to another organism.

RNA-sequencing results were trimmed and aligned to the *Mus musculus* genome, version mm39 (PRJNA169), using HISAT2^31^^,^^32^ on Partek Genomics Flow software, v.11.0 (St. Louis, MO, USA), followed by differential gene expression analysis using edgeR.^33^^,^^34^^,^^35^ A multidimensional scaling plot showed clustering for samples at different ZTs and mosquito-bite status, suggesting a distinct gene expression response between these groups (Figure 2A). Volcano plots show a pairwise comparison of the differentially expressed genes after mosquito bite among the different groups at ≤0.05 false discovery rate cutoff (Figure 2B). At ≥2 log_2_FC cutoff, ZT11 exhibited the greatest number of differentially expressed genes at 117. In contrast, ZT1 only yielded 23 differentially expressed genes. The complete list of genes is summarized in Table S3. A heatmap to depict all genes showing the dynamics of gene expression between these time points is shown in Figure S1.

Among these two groups, there are 18 genes that overlap (Figure 2C). All these genes are upregulated in response to a mosquito bite, as shown in the heatmap (Figure 2D). Using gene-ontology (GO) enrichment analysis,^36^^,^^37^^,^^38^^,^^39^ these genes are enriched in the categories of inflammatory, defense, and stress responses (Figure 2E).

Interestingly, there are only five genes that are uniquely expressed at ZT1. *Slc13a3*, upregulated at 2.2 log_2_FC, is a sodium-dicarboxylate cotransporter. *Lilra6* is a leukocyte immmunoglobulin-like receptor A6 and is induced at 2.1 log_2_FC. *Epithelial mitogen* is a member of the epidermal growth factor family that is involved in cell survival, proliferation, and migration, and is induced at 2.5 log_2_FC. Interestingly, *viperin* is also induced upon mosquito bite, at 2.04 log_2_FC. This is an interferon-inducible antiviral protein that plays a role in in cellular antiviral response and innate immune signaling. Finally, *Ifi205,* which is upregulated at 2 log_2_FC, is another interferon-inducible genes that have not been well characterized.

### Mosquito bite elicited gene expression for immune cell migration and defense against pathogen

**Figure 3:**
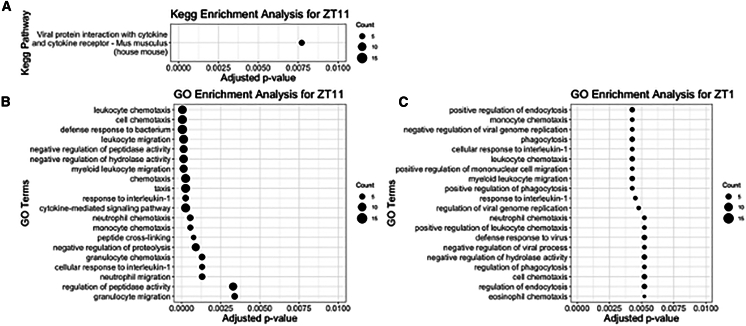
KEGG and GO-term analysis for all upregulated DEGs across all time points (A) KEGG pathway highlighted the role for mosquito salivary proteins to elicit viral responses. (B and C) The top 20 GO-term enrichment for biological processes are shown for (B) ZT11 and (C) ZT1 time point, highlighting the role for mosquito salivary proteins to elicit immune cell response.

We further focused on the list of upregulated genes and performed Kyoto Encyclopedia of Genes and Genomes (KEGG) pathway and GO-term analysis for all examined timepoints. KEGG pathway analysis showed that at ZT11, murine skin exhibited a strong response toward viral protein interactions, involving cytokines and cytokine-receptor pathways (Figure 3A). GO-term analysis indicated that most genes that are upregulated upon mosquito bite at ZT11 are in the category of immune genes that are associated with infection (Figure 3B). For example, genes that link to the migration and chemotaxis of different immune cells, such as leukocyte, neutrophil, granulocyte, monocyte, lymphocyte, myeloid leukocyte, and eosinophil, are upregulated. In addition, genes related to a defense response to bacterium, such as cytokine-mediated signaling pathways and cellular response to IFN-beta, are also upregulated. Similarly, we also observed genes that are related with migration and chemotaxis of different immune cells, such as leukocyte, neutrophil, granulocyte, monocyte, lymphocyte, myeloid leukocyte, and eosinophil, are upregulated in ZT1 (Figure 3C).

### Mosquito bite elicited a similar trend in differential gene expression in different area of skin

**Figure 4:**
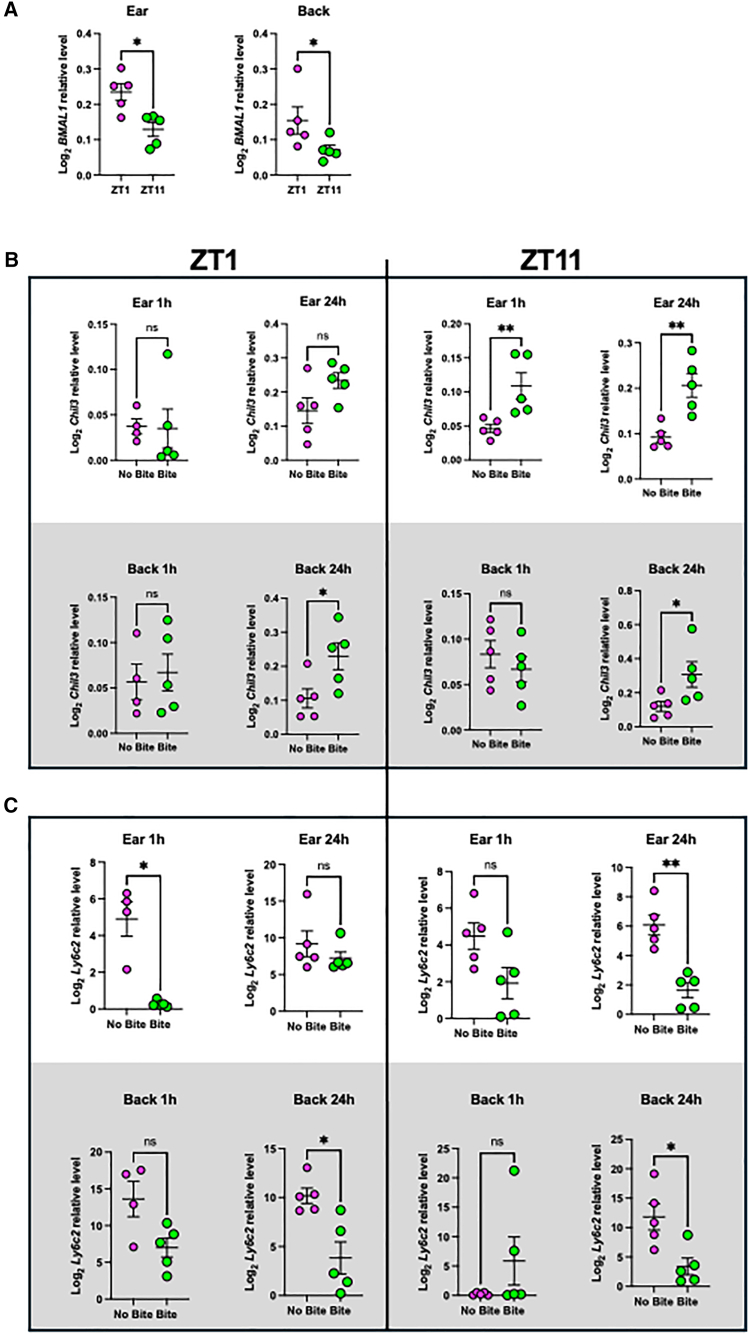
Murine skin showed differential gene expression of similar trend upon mosquito bite after 1 h and 24 h at both ZT1 and ZT11 (A) *BMAL1* relative expression of mice ear skin and back skin were verified to have high expression at ZT1 and low expression at ZT11. (B) *Chil3* relative expression of mice ear skin and back skin were examined after 1 h and 24 h of mosquito bite at ZT1 and ZT11. (C) *Ly6c2* relative expression of mice ear skin and back skin were examined after 1 h and 24 h of mosquito bite at ZT1 and ZT11. Statistical significances were measured using Mann-Whitney *t* test; ∗*p* < 0.05 and ∗∗*p* < 0.01; error bars represent standard error of the mean (SEM).

We selected two genes that are involved in inflammatory responses from Figure 2 for further assessment by qPCR, which are Chil3 and Ly6c2. We collected skin from the ear and the back of the mice upon mosquito bite at ZT1 and ZT11 after 1 h and 24 h of exposure to mosquito bite. In general, Chil3 showed a trend toward upregulation of gene expression across all time points and tissue examined, with statistical significance observed at ZT11 after both 1 h and 24 h of exposure on the ear skin, and after 24 h of exposure on the back skin at both ZT1 and ZT11 (Figure 4B). Interestingly, Ly6c2 showed a general downregulation trend in gene expression with statistical significance observed at 24 h in the ear skin at ZT11, and in the back skin at both ZT1 and ZT11 (Figure 4C). Overall, we observed a similar trend in differential gene expression upon mosquito bite when examined in different area of the skin and after 1 h or 24 h of exposure.

### Mosquito bite at ZT11 resulted in higher number of immune cells in the skin

**Figure 5:**
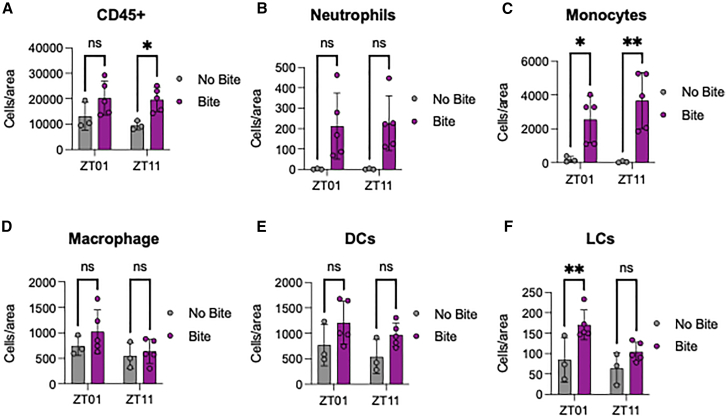
Mosquito bite induced more immune cells on the skin (A) Overall immune cell types were examined on the mice ear after 24 h of mosquito bite. (B–F) Specific immune cells were examined for (B) neutrophils, (C) monocytes, (D) macrophages, (E), dendritic cells, and (F) Langerhans cells. Multiple comparison two-way ANOVA were performed to examine statistical significance between no bite and bite group of each ZT; ∗*p* < 0.05 and ∗∗*p* < 0.01; error bars represent standard error of the mean (SEM).

To understand the effect of the mosquito bites on the immune cells, we measured the number of immune cells on the skin at 24 h after mosquito bite. Indeed, there are more immune cells as depicted by CD45 positive cells at ZT11 (Figure 5A). Additionally, we observed a statistically significant increase in monocytes cell number at both time points, but not any other cell type examined (Figure 5C). Interestingly, significant upregulation of Langerhans cells was only observed at ZT1 (Figure 5F).

Overall, these data indicate a stronger response to mosquito bites at ZT11 compared with ZT1. High number of genes associated with immunity and infection response that are upregulated at ZT11, suggesting a well-regulated immune and cellular response at this time of day. Since ZT11 marks the beginning of the active phase in mice, which corresponds to the morning time in humans, it suggests that the immune system is most prepared to respond to infections during this period. In contrast, ZT1 exhibited limited number of differentially regulated genes regulation, which closely reflecting a resting state.

## Discussion

This study examined the influence of diurnal rhythm on the murine immune response to mosquito bites. We found that mosquito bites generally changed the expression of genes involved in adaptive immunity 24 h post-bite. The degree of transcriptional response varied with the time of the day, with ZT11 showing the most gene dysregulation, at 117 genes. Only 23 gene expression changes were noted at ZT1, with 18 overlapping between ZT1 and ZT11 (Figure 2C). Consistent with previous findings, most effected genes are related to immunity pathways that are involved in inducing inflammation or recruitment of immune cells to the bite site.^40^^,^^41^ The number of differentially expressed genes in response to a mosquito bite is albeit lower than previous findings of over 400 genes affected after 48 h in human,^25^ which is likely due to differences in cutaneous site, species, and time of the day. Indeed, in humans, skin surface parameters such as sebum excretion, skin temperature, and *trans*-epidermal water loss exhibited distinct rhythmicity between different sites such as the face and forearm.^42^ Our findings underscore the importance of diurnal rhythm in assessing the impact of mosquito bites on skin cells.

Migration of leukocytes,^4^^,^^5^ dendritic cells,^3^ and neutrophils^6^ from the skin to the lymph nodes has been shown to exhibit circadian oscillation. This group of immune cells can also be affected by mosquito salivary proteins^40^^,^^43^ and targeted by arboviruses disseminated by *Aedes* sp. mosquitoes, such as dengue,^44^^,^^45^ Zika,^46^ and chikungunya virus.^47^ Further, mosquito salivary proteins can induce local inflammation that promotes recruitment of immune cells to the bite site, rendering them susceptible to arboviral infection,^48^ the precursor of systemic infection. For example, *A. aegypti* neutrophil recruitment protein present in the mosquito saliva promotes an influx of neutrophil and virus-susceptible myeloid cells to the bite site and facilitates local and systemic infection by Zika and dengue viruses.^41^ Further, the *A. aegypti* venom allergen-1 protein can enhance infection of flaviviruses to the monocytes by activating the autophagy pathway, which is a common pathway employed by flaviviruses to enter the cells and subsequently initiate cellular infection.^49^

Consistently, we found genes that are associated with neutrophil, monocyte, granulocyte, eosinophil, and lymphocyte migration and chemotaxis to be differentially regulated in adult mice at ZT11 and ZT1 upon mosquito bite (Figure 3). However, the number of differentially expressed genes is lower at ZT1, suggesting that this process may be inefficient during this time point. Previous studies on evaluation of immune cells infiltrate to the mosquito bite site did not account for the diurnal rhythm factor, and comparing results from different studies have been challenging as there are discrepancies in the type of immune cells that infiltrate the site of mosquito salivary gland to extract inoculation, either by natural mosquito biting^43^ or by needle injection.^40^ Our examination of immune cell population in the skin showed significant upregulation of immune cells at ZT11, corroborating our hypothesis that there are differences in the magnitude of responses that are tied to diurnal rhythm. Interestingly, we also observed significant increase of Langerhans cells at ZT1, but not at ZT11, further underscoring the effect of diurnal rhythm on cutaneous responses to the mosquito blood-feeding. This is of significant interest as Langerhans cells is among the early target for flavivirus infection in the dermis, such as dengue virus^50^^,^^51^ and Zika virus.^46^ It remains to be seen whether infection by vector-delivered arboviruses will also be influenced by the diurnal rhythm, which may further emphasize the importance of the diurnal rhythm in studying vector-borne diseases.

We also examined the effects of mosquito bite at different times of day on innate immune gene expression. Previously we have shown that components of the innate immune response, namely, toll-like receptors (TLRs), exhibited circadian and diurnal pattern of gene expression in the spleen.^52^ In addition, *TLR3*, but not *TLR7*, was shown to be downregulated at 4 h following mosquito-delivered chikungunya virus infection.^53^ We quantified TLR2, TLR7, and TLR9 gene expressions by qPCR and detected no changes in gene expression upon mosquito bite after 24 h(data not shown). This may be attributed to the timing of the biopsy harvest at 24 h after mosquito bite. Guerrero et al. suggested that the dynamic of cutaneous immune response upon mosquito bite begins with innate immune responses after 4 h, and later shifts toward adaptive immune responses after 48 h.^25^ Similarly, we did not detect differences in other innate immunity genes such as the antiviral proteins and antimicrobial peptide gene expression^8^^,^^54^ (Table S3).

Little is known about the regulation of the diurnal rhythm that governs blood-feeding activity in mosquitoes. *A. aegypti*, an anthropophilic mosquito species, prefers to blood-feed during the daytime, with the peak of host-seeking activity occurring at dawn and dusk.^55^ Moreover, exposure to artificial light during the subjective night has been shown to increase mosquito biting behavior.^56^ A recent study demonstrated that *A. aegypti* exhibits a stronger preference for light at ZT11,^57^ indicating heightened activity that may correlate with increased biting behavior. These observations may also partially explain our findings of elevated immune responses at mosquito-bite sites at ZT11 compared with ZT1.

The timing of biopsy collection is crucial in understanding gene-expression effects, as prior studies have shown that mosquito bites can cause differential gene expression and protein activity ranging from 30 min to 7 days on the skin.^25^^,^^40^^,^^43^ In addition, distinct mosquito salivary proteins affect specific cell types and may therefore cause differential effects during the circadian cycle. Overall, this study offers a glimpse into the importance of accounting for the diurnal rhythm when studying host-vector interactions.

### Limitations of the study

This study provides important insights into the influence of diurnal rhythms on immune responses to mosquito bites. However, several limitations should be considered. First, the analysis was restricted to two timepoints (ZT1 and ZT11), which may not encompass the full spectrum of circadian variations in immune activity. While shifts in immune cell populations were reported, further functional investigations are necessary to elucidate the downstream implications of these changes. It is also critical to recognize that structural and circadian differences between murine and human skin may affect the broader applicability of these results. Finally, although the study discusses the role of mosquito salivary proteins, their specific individual effects were not delineated.

## Resource availability

### Lead contact

Further information and requests for resources and reagents should be directed to and will be fulfilled by the lead contact, Hamidah Raduwan (hamidah.raduwan@yale.edu).

### Materials availability

The study did not generate new materials.

### Data and code availability

• RNA-sequencing data supporting this study are available at ArrayExpress—Functional Genomics Data under EMBL’s European Bioinformatics Institute with the accession number E-MTAB-15581.
• This study did not generate any original code.
• Any additional information required to re-analyze the data reported in this paper is available from the lead contact upon request.

## Acknowledgments

This work was supported in part by the 10.13039/100000002NIH (AI142624) and the Howard Hughes Medical Institute Emerging Pathogens Initiative. The Yale Center for Genomics Analysis (YCGA) sequencing core at which the samples are sequenced is supported by the 10.13039/100019941HPC grant (1S10OD030363-01A1).

## Author contributions

H.R. and E.F. conceived and designed the experiments with R.R.M. and A.C.S. H.R., A.M.-L., M.H.S., and T.-Y.C. performed the experiments. H.R. and J.P. analyzed the RNA-seq data. H.R. and E.F. wrote the manuscript with contributions from all authors. All authors have approved the final version of the manuscript.

## Declaration of interests

The authors declare no competing interests.

## Declaration of generative AI and AI-assisted technologies in the writing process

During the preparation of this work, the authors used ChatGPT to assist with improving clarity, readability, and grammar. After using this tool/service, the authors reviewed and edited the content as needed and take full responsibility for the content of the publication.

## STAR★Methods

### Key resources table

**Table:** 

REAGENT or RESOURCE	SOURCE	IDENTIFIER
**Biological samples**		

*Aedes aegypti* Orlando strain	Dr. Erol Fikrig Laboratory	N/A
*Mus musculus* C57BL/6J	Jackson Laboratory	000664

**Biological and chemical reagents**		

RNeasy fibrous tissue mini kit	Qiagen	Cat# 74704
iScript™ cDNA Synthesis Kit	Biorad	Cat# 1708891
iTaq Universal SYBR Green Supermix	Biorad	Cat# 1725121
Oligonucleotides	The Keck Oligonucleotide Synthesis facility	See Table S5
Liberase TM	Millipore-Sigma	Cat# 5401119001
Percoll	Cytiva	Cat# 17089102
APC α-CD45.2, clone 104	Biolegend	Cat# 109813; RRID:AB_389210
AF700 α-CD3	Biolegend	Cat# 152316; RRID:AB_2732713
AF700 α-CD5	Biolegend	Cat# 100636; RRID:AB_2687002
AF488 α-Ly6G	Biolegend	Cat# 127626; RRID:AB_2561340
PE α-CD64	Biolegend	Cat# 139304; RRID:AB_10612740
PerCPcy5.5 α-EpCam	Biolegend	Cat# 118220; RRID:AB_2246499
PEcy7 α-CD11c	Biolegend	Cat# 117318; RRID:AB_493568
PacBlue α-CD11b	Biolegend	Cat# 101224; RRID:AB_755986
BV605 α-Ly6C	Biolegend	Cat# 128036; RRID:AB_2562353
fixable viability dye eFluor 506	Thermo Fisher Scientific	Cat# 65-0866-18
Accucheck cell counting bead	Invitrogen	PCB100

**Deposited data**		

*Mus musculus* RNA-seq data	This study	EMBL-EBI: E-MTAB-15581
*Mus musculus* genome mm39	NCBI	NCBI: PRJNA169

**Software and algorithms**		

Partek^TM^ Genomics Flow software, v11.0	Illumina	https://www.illumina.com/products/by-type/informatics-products/partek-flow.html
edgeR (v4.2.1)	Bioconductor	https://bioconductor.org/packages/release/bioc/html/edgeR.html
ComplexHeatmap (v2.16.0)	Bioconductor	https://www.bioconductor.org/packages/release/bioc/html/ComplexHeatmap.html
R version 4.4.1	R	https://www.r-project.org/
clusterProfiler (v4.12.5)	Bioconductor	https://bioconductor.org/packages/release/bioc/html/clusterProfiler.html
Prism v10	GraphPad	www.graphpad.com/

### Experimental methods and study participant details

#### Mosquitoes

*Ae. aegypti* (Orlando strain) mosquitoes were maintained in a climate-controlled room at 28 °C with a relative humidity ranging between 60 and 80%, following a light:dark cycle of 14:10 h. Upon hatching, larvae were separated into pans at an approximate density of 200 larvae per pan and were provided with fish food (WardleyAquatics). Adult mosquitoes had *ad libitum* access to cotton rolls soaked in a 10% sucrose solution. The mosquito colonies were sustained by feeding female mosquitoes on blood sourced from naive AG129 mice (IACUC202110404).

#### Mouse

Female C57BL/6J mice (6–8 weeks) were ordered from Jackson laboratory. Mice were allowed to synchronize on a 12:12h light and dark cycle for at least one week prior to mosquito blood feeding. The mice had *ad libitum* access to food and water throughout the entire study. All mice are housed and maintained in the vivarium following an approved Yale IACUC protocol (IACUC202110404).

### Method details

#### Mosquito blood feeding

Mosquito colonies were evenly divided to groups and 20 mosquitoes were allowed to bite on the mouse ear at the respective time point for about 30min. The mosquito bite sites were then marked with surgical skin marker. 24 h later, 3.5 mm ear punch biopsies were taken. Tissues are collected into RLT buffer (Qiagen Cat#79216) and immediately subjected to RNA extraction as described below.

#### RNA extraction and sequencing

RNA extraction was performed from murine skin using the RNeasy fibrous tissue mini kit (Qiagen 74704) following the manufacturer’s protocol. Briefly, skin tissues were homogenized using a KIMBLE pestle inside a centrifuge tube for 30s-1min or until most tissues were lysed. Proteinase K was then added, and tissues were incubated in a 55°C water bath for 10min. The mixture was spun at 10,000g for 5min and supernatant collected in a new tube. Ethanol was added to the supernatant, gently pipetted for mixing, and transferred to an RNeasy Mini column. Columns were spun to allow RNA to bind to the silica, washed with RW1, RDD, and RPE buffer per manufacturer’s protocol. Samples were eluted with 30 L RNAse-free water and subjected to RNA-sequencing. RNA libraries were prepared by removal of ribosomal RNA and sequenced using an Illumina HiSeq 2500 instrument by generating paired end reads at 150 bp length at the Yale Center for Genome Analysis (YCGA).

#### Differential gene expression analysis

RNA-seq analyses including trimming of adapter sequences, alignment, and gene-level annotation were performed using Partek Genomics Flow software, v11.0 (St. Louis, MO, USA). Specifically, RNA-seq data were trimmed and aligned to the *Mus musculus* genome, version mm39 (PRJNA169) using HISAT2.^31^^,^^32^ The gene-level data were normalized using likelihood ratio test in edgeR (v4.2.1). Figures were generated using ggplot2 (v3.5.1). DEGs were considered significant if glmLRT-estimated shrunken log_2_FC was <2 or >2 and with a false discovery rate <0.05. Multidimensional plot was generated using EdgeR, heatmaps were prepared using ComplexHeatmap (v2.16.0). All DEGs analysis post gene-level annotation were performed on R version 4.4.1. GO and KEGG term analysis were performed on clusterProfiler (v4.12.5). The transcriptomic data is available in the Gene Expression Omnibus repository at the National Center for Biotechnology Information (GSE annotation number will be provided upon acceptance of the manuscript).

#### Quantitative PCR

cDNA was generated with the iScript cDNA Synthesis Kit (Bio-Rad) following the manufacturer’s protocol using 250 ng of RNA per reaction. Quantitative real-time PCR was performed using iTaq Universal SYBR Green Supermix (BioRad) per manufacturer’s protocol on Bio-Rad CFX96 Real-Time System. Relative quantification of gene expression levels were performed with GAPDH as the reference gene. A complete list of the oligonucleotide sequence is in Table S5.

#### Isolation of immune cells from mouse skin

Mice were euthanized and ear pinnae excised and plunged into ice-cold PBS. Ventral and dorsal dermal sheets were separated, and biopsies were minced and placed in digestion buffer (RPMI, 0.25 mg/mL Liberase TM, 0.5 mg/mL DNase). Minced biopsies were digested for 45 min at 37°C with gentle shaking at 180RPM. Following digestion, the suspension was passed through a 70 m cell strainer with the aid of a 3 mL syringe plunger. Cells were washed twice (RPMI 5% FBS) before immunocytes were enriched via a single 35% Percoll gradient.

#### Flow cytometric analysis

Isolated immune cells were stained for 20 min at 4°C with the following antibody cocktail diluted into FACS buffer (PBS 2% FBS): APC α-CD45 (clone: 1:400), AF700 α-CD3 (1:400; Biolegend 152316), AF700 α-CD5 (1:400; Biolegend 100636), AF488 α-Ly6G (1:400; Biolegend 127626), PE α-CD64 (1:400; Biolegend 139304), PerCPcy5.5 α-EpCam (1:400; Biolegend 118220), PEcy7 α-CD11c (1:400; Biolegend 117318), PacBlue α-CD11b (1:400; Biolegend 101224), BV605 α-Ly6C (1:400; Biolgend 128036) and fixable viability dye eFluor 506 (1:1000; ThermoFisher 65-0866-18). Cells were washed and fixed with BD Cytofix/Cytoperm (BD 554714) for later analysis. AccuCheck cell counting beads (Invitrogen PCB100) were added to all samples for cell number quantification. Calculated cell numbers were then normalized to biopsy area. All flow cytometry data was collected on BD LSR2 with FACSDiva 7 software. Flow cytometry data were analyzed with FlowJo (v10.10.0).

### Quantification and statistical analysis

The analysis of all data was performed using the unpaired Welch’s t-test, nonparametric Mann–Whitney t-test, or two-ways ANOVA as specified in figure legend using Prism 10 software (GraphPad Software, Inc, San Diego, CA). A *p*-value of <0.05 was considered statistically significant, with ∗ equals to *p* < 0.05 and ∗∗ equals to *p* < 0.01.

## Supplemental information

Document S1. Figures S1 and S2 and Table S5

Table S3. The raw count per million values for the complete list of genes that are differentially regulated for all sample replicates The raw counts per million (CPM) value representing all sample replicates that were shown to be significant are listed in this table.

Table S4. The complete log2FC value for all genes that are differentially regulated Differentially expressed genes that meet the cutoff of ≤0.05 false discovery rate (FDR) and ≥ 2-fold log_2_FC or ≤ 2-fold log_2_FC are plotted.
