# Supplementary material for: The cutaneous response to a mosquito bite is influenced by the diurnal rhythm
Source: iScience. 2025 Sep 30;28(11):113666. doi: 10.1016/j.isci.2025.113666 (PMC12554125; doi:10.1016/j.isci.2025.113666)
Supplement: Document S1. Figures S1 and S2 and Table S5 [file mmc1.pdf]

iScience, Volume *28*

Supplemental information

The cutaneous response to a mosquito bite

is influenced by the diurnal rhythm

Hamidah Raduwan, Jinhee Park, Alejandro Marín-López, Mathias H. Skadow, Tse-Yu Chen, Richard A. Flavell, Albert C. Shaw, Ruth R. Montgomery, and Erol Fikrig

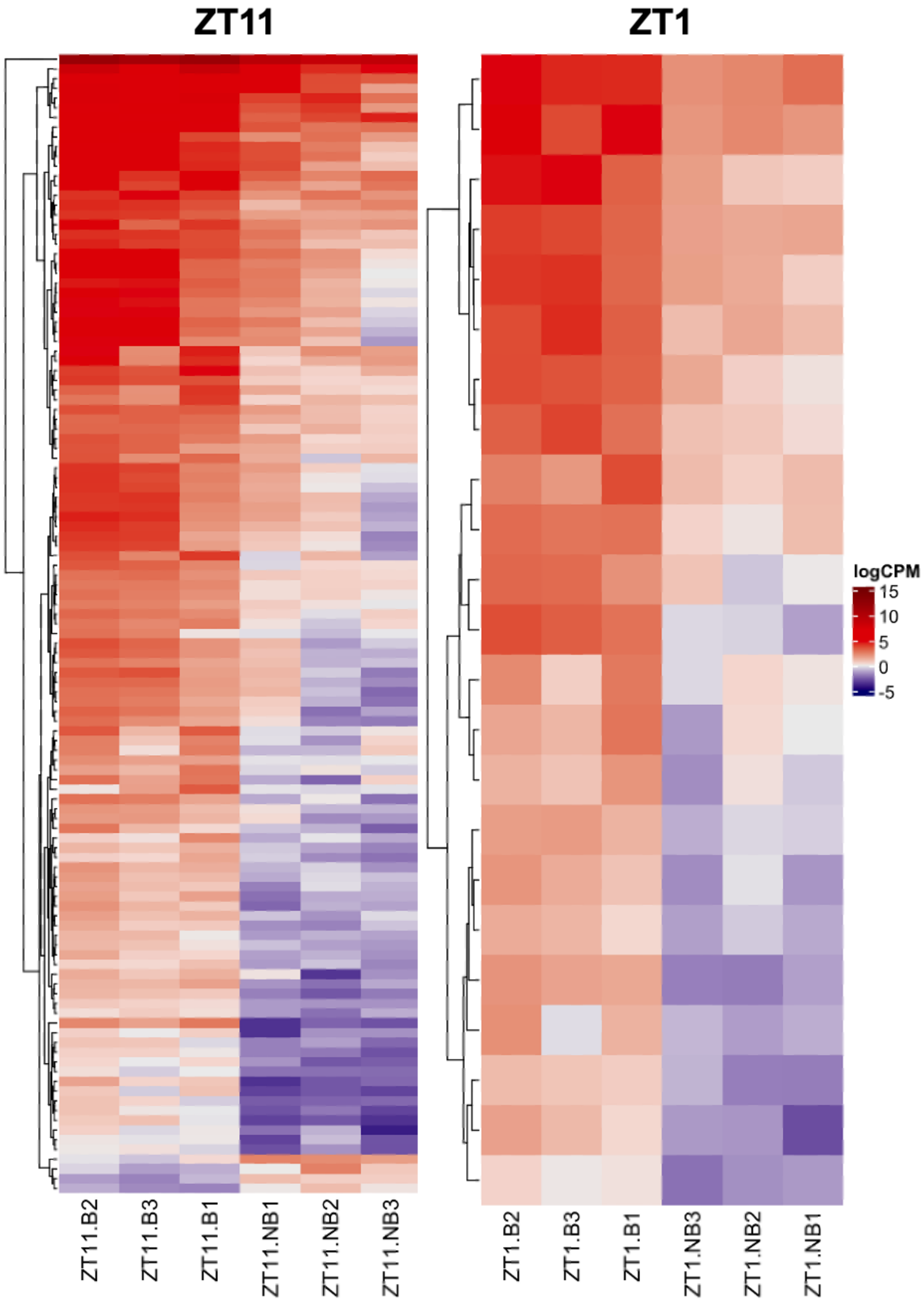

Figure S1. Heatmap of all significant DEGs across all time points. Differentially expressed genes that meet the cutoff of ≤ 0.05 false discovery rate (FDR) and ≥ 2-fold $\log_2$FC or ≤ 2-fold $\log_2$FC are plotted.

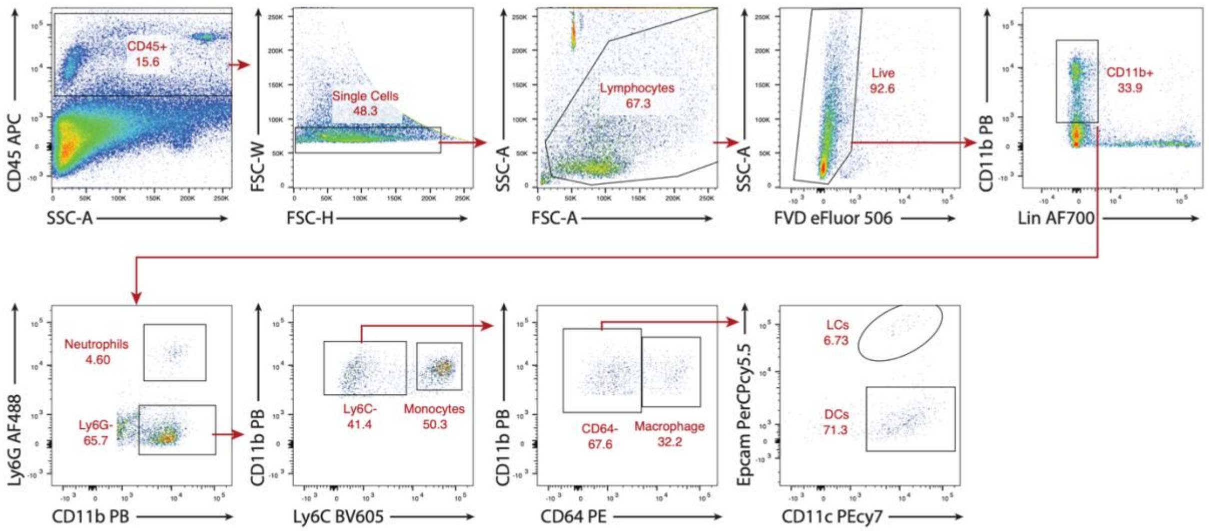

Figure S2. Gating strategy flow cytometry. Cells were collected from mice ear biopsies and ran on flow cytometry to identify different types of immune cells that were affected by mosquito blood feeding.

Genes	Direction	Sequence
BMAL1	Forward	CTCAACCATCAGCGACTTCATG
	Reverse	TGCCTTTCCTCTTGCGATTG
GAPDH	Forward	AGGTCGGAGTCAACGGATTTG
	Reverse	GGTCATTGATGGCAACAATATCCA
Chil3	Forward	TCTGAATGAAGGAGCCACTGAG
	Reverse	CACGGCACCTCCTAAATTGTTG
Ly6c2	Forward	ATCTCTATTCTTGGCCCTGGAG
	Reverse	TCATTGACAGGCACACATGG

Table S5. The list of oligonucleotide sequences used in the study
